# The “Great Family” of Cardiovascular Scientific Journals in Brazil

**DOI:** 10.21470/1678-9741-1-2020-0608

**Published:** 2020

**Authors:** Paulo Roberto B. Evora

**Affiliations:** 1 Department of Surgery and Anatomy Ribeirão Preto School of Medicine. Faculdade de Medicina de Ribeirão Preto da Universidade de São Paulo (FMRP-USP), Ribeirão Preto, SP, Brazil.

The main objective of a scientific society is to promote dissemination of our best science by journals of increasingly high quality, so that it can attract deserving attention and to make a more influential contribution to the world scientific community. Undoubtedly, the proximity of the journals in a “family” model allows a greater success of each journal individually. This has undoubtedly been a trend in world science, particularly in Cardiology, as exampled by the impressive growth of the American College of Cardiology (JACC) family of journals, associated with the US American College of Cardiology, and the European Heart Journal (EHJ) family, associated with the European Society of Cardiology (ESC) in Europe^[1,2]^.

I am a testimony that the “Great Family” of Cardiovascular Scientific Journals in Brazil was a Dr. Carlos Rochitte dream during our role as Associate Editors for *ABC Cardiol (Brazilian Archives of Cardiology)*, under the leadership of Editor-in-Chief Luis Felipe Moreira. Besides our significant decisions in adopting only the electronic publication in English language, Dr. Rochitte always insisted on the “great family idea”. We adopted the first association with the *International Journal of Cardiovascular Sciences*.

At that time, we had a great and unknown concern about a possible negative interference between different journals. This feeling was reinforced because the editorial policy of the *ABC Cardiol* has progressively privileged publications focused on clinical aspects. This fact was also associated with the creation of *Revista Brasileira de Cirurgia Cardiovascular (Brazilian Journal of Cardiovascular Surgery, BJCVS)*, specifically directed to the surgical aspects of heart diseases, which began to be published on August 1986^[3]^. Invited by Dr. Luis Felipe Moreira, I wrote an Editorial about the impact of the creation of BJCVS on the profile of publications with respect to cardiac surgery issues in *ABC Cardiol*. Querying MEDLINE in the period 1983-1985 (before BJCVS), the following numbers were calculated: a total of 615 studies, 58 (9.43%) about heart surgery. As a final comment, we concluded that the studies published in *ABC Cardiol* may be considered compatible with the orientation to disseminate information of mutual interest between physicians and surgeons^[4]^.

The great family of cardiology journals will be useful in publishing good articles. Often good articles are rejected or have unacceptable long publication times, a situation created by the great demand for submissions. Case Reports, now blocked, will again have their deserved importance, since there are several reasons to be “kept alive”^[5]^ It is necessary that all “family members” maintain hyperactivity around two basic parameters for authors: indexing in MEDLINE and the search for increasing impact factors. It would be appropriate to highlight some points directly and indirectly involved with the impact factor: a) High publication fees; b) The so-called “research consortium”, with papers that have up to 200 co-authors; c) Often abusive self-citation rates; and d) The billionaire new mergers of editorial groups, etc.^[6]^.

*Either as a family or as “friends”, I believe that an effective collaboration between nationally renowned journals allows the growth of a group of journals that would not be seen by the journals alone. Hopefully, this initial and preliminary collaboration will lead to a broader discussion and closer interaction between journals with similar aims and scope.*

*(Carlos Eduardo Rochitte, 2020)*
